# Fœtus Compressus

**Published:** 1885-12

**Authors:** Miles F. Porter

**Affiliations:** Fort Wayne, Indiana


### FŒTUS COMPRESSUS.

TO THE EDITORS OF THE *Chicago Medical Journal and Examiner.*

*Gentlemen:*

On April 19th, 1879, I was called to attend Mrs. P., 25 years old, of nervous temperament, healthy, good family history, in her first confinement. After a somewhat protracted, though normal labour, she was delivered of a fairly nourished female child, weighing eight pounds. Immediately preceding the expulsion of the placenta, which followed a few minutes after the birth of the child, a four months' *fœtus compressus* was expelled. The head of the fœtus was flattened from side to side and turned with the chin to the shoulder, while the body was flattened from before backward. The placenta was not examined closely. The mother had some pain and hæmorrhage at the fourth month, which was controlled by rest and opium. She was fully as large at four as at six months, growth seeming to stop after these symptoms until the latter months of pregnancy. The living child never seemed robust and died at the age of three months, from whooping-cough. The mother has been delivered of three children since,—all normal labours,—and all the children are living and healthy at the present time. The *fœtus* was a male.

MILES F. PORTER, M. D.

Fort Wayne, Indiana.

